# Removal of Organic Compounds with an Amino Group during the Nanofiltration Process

**DOI:** 10.3390/membranes12010058

**Published:** 2021-12-31

**Authors:** Renata Żyłła, Magdalena Foszpańczyk, Magdalena Olak-Kucharczyk, Joanna Marszałek, Stanisław Ledakowicz

**Affiliations:** 1 Łukasiewicz Research Network-Textile Research Institute, Brzezińska 5/15 Str., 92-103 Łódź, Poland; magdalena.foszpanczyk@iw.lukasiewicz.gov.pl (M.F.); magdalena.olak-kucharczyk@iw.lukasiewicz.gov.pl (M.O.-K.); 2 Department of Environmental Engineering, Faculty of Process and Environmental Engineering, Lodz University of Technology, Wólczańska 213 Str., 90-924 Łódź, Poland; joanna.marszalek@p.lodz.pl; 3 Department of Bioprocess Engineering, Faculty of Process and Environmental Engineering, Lodz University of Technology, Wólczańska 213 Str., 90-924 Łódź, Poland; stanislaw.ledakowicz@p.lodz.pl

**Keywords:** nanofiltration, amines, hazardous compounds

## Abstract

The research covered the process of nanofiltration of low molecular weight organic compounds in aqueous solution. The article presents the results of experiments on membrane filtration of compounds containing amino groups in the aromatic ring and comparing them with the results for compounds without amino groups. The research was carried out for several commercial polymer membranes: HL, TS40, TS80, DL from various manufacturers. It has been shown that the presence of the amino group and its position in relation to the carboxyl group in the molecule affects the retention in the nanofiltration process. The research also included the oxidation products of selected pharmaceuticals. It has been shown that 4-Amino-3,5-dichlorophenol—a oxidation product of diclofenac and 4-ethylbenzaldehyde—a oxidation product of IBU, show poor separation efficiency on the selected commercial membranes, regardless of the pH value and the presence of natural organic matter (NOM). It has been shown that pre-ozonation of natural river water can improve the retention of pollutants removed.

## 1. Introduction

The recent developments in more advanced analytical methods have revealed new threats associated with the presence of various micropollutants in drinking water sources. One of such micro-pollutants are N-nitrosamines (NA). In many industrialized countries, they attract attention because of their high carcinogenicity and frequent occurrence in disinfected drinking water, in which chemical compounds initiating their formation are used [1]. Bei et al. [2] tested approximately 164 samples of drinking water intended for human consumption in China for the presence of NA. They found that NA concentration in water was much higher in China than in the United States. Nine N-nitrosamines were detected, among which the carcinogenic N-nitrosodimethylamine (NDMA) had the highest concentration. Several laboratory and pilot studies were carried out to determine factors that affect the increase or decrease of NDMA concentration in drinking water during water treatment [3]. It has been found that there are many precursors from which NA can be formed during water treatment (e.g., in the process of chlorination and even ozonation) [3,4]. Among such precursors there are aromatic amines, which, themselves, may be mutagenic, carcinogenic and toxic [5,6]. Recent studies have shown that pharmaceuticals and personal care products (PPCPs) with substituted amino groups can be precursors for NDMA formation during disinfection with chloramine [7,8]. The best-known pharmaceuticals recognized as precursors of N-nitrosamines are ranitidine and nizatidine [9,10,11].

As recently assessed by Platzek [12], exposure to the presence of aromatic amines (AAs) in consumer products carries a risk to human health, especially related to the mutagenic and/or carcinogenic properties of some AAs. The toxicity of AAs depends on the metabolic activation of an amino group that can produce reactive intermediate hydroxylamine, which is known to damage DNA and proteins [13].

Filtration processes are widely used in many areas of life, in the food and pharmaceutical industries, as well as in environmental protection [14,15,16,17,18]. The effectiveness of retention of a given compound in the membrane filtration process depends on many factors, for example, structure, charge and the molecular weight of the compound being removed, and its affinity for the membrane, as well as its chemical structure and membrane properties. The chemical composition of the water matrix can significantly affect the effectiveness of the separation process. Most of the research is carried out on pure aqueous solutions. Nevertheless, the presence of n-nitrosamine precursors is mainly related to surface water (lakes and rivers).

Surface waters, which are the main resources of drinking water in many parts of the world, contain a number of pollutants, including natural organic matter (NOM). NOM is a complex matrix of organic matter derived from the decomposition of living terrestrial and aquatic organisms and consists mainly of humic and fulvic and non-humic fractions, which are a mixture of carbohydrates, amino acids, and proteins [19,20]. The presence of organic matter in an aqueous solution may cause severe membrane contamination, which can lead to a decrease in the filtrate flux and reduce retention efficiency [20,21,22].

The removal effectiveness of the compounds containing an amino group in their structure during nanofiltration process depends on the additional functional groups in the molecule. Amino acids that have, in addition to the amino group, also a carboxyl group may have a different charge depending on their pH [23,24]. This is often used to separate a mixture of different amino acids in a membrane filtration process [25]. High separation efficiency is obtained when the membrane has the same surface charge as the amino acid [24]. Therefore, the pH of the water matrix is important. Polyamide nanofiltration membranes exhibit negative surface potential in contact with a solution of normal pH because of the predominance of the unreacted carboxylic acid group in comparison with the amine groups in the membrane. However, with a change in the pH of the solution there will be protonation or de-protonation of the functional groups of the membranes that results in the change of the membrane surface potential [26]. At the isoelectric point the net surface potential of nanofiltration membranes becomes zero, whereas the membranes show a positive surface potential below the isoelectric point and negative potential above it [26].

The aim of the study was to investigate the effect of the presence of amino groups on the effectiveness of nanofiltration. The study also includes tests for selected oxidation products of diclofenac (DCF) and ibuprofen (IBU). Due to the serious risk associated with the emission of amines to the environment, such tests are justified. The article does not cover all the needs related to the lack of knowledge in the field of degradation of aromatic amines and some pharmaceutical oxidation products. However, it makes an important contribution to the field of nanofiltration and the chemistry of hazardous environmental pollutants.

## 2. Methodology

### 2.1. Materials

Twelve compounds were tested in the study: amines, amino acids and pharmaceutical oxidation products. Table 1 shows the structural formulas of the above-mentioned compounds and their basic properties.

Ibuprofen and diclofenac oxidation products: 4-Amino-3,5-dichlorofenol (4-A3,5-Cl), sodium salt 2-Aminophenylacetic acid (2-APA) and 1-(4-isobutyl-phenyl)ethanol (MPPE) were synthesized and provided by SIA Chemspace (Riga, Latvia). Ibuprofen (IBU) sodium salt, salicylic acid (SA), acetylsalicylic acid (ASA), 4-ethylbenzaldehyde (4-EBA), p-aminobenzoic acid (PABA), phenyloacetic acid (PAA), 5-amino-o-kresol (KR) and anthranilic acid (AA) were purchased from Sigma Aldrich (Poznań, Poland), and 1-hydroxyibuprofen (OH-IBU) was obtained from AKos GmbH (Lörrach, Germany). For the tests, solutions with a mass concentration of the active substance of 5 mg·L^−1^ were used. The selected compounds were dissolved in pure deionized water or, if the experiment required it, in natural raw or ozonated (15 min) water from rivers.

### 2.2. Research Equipment

**Figure 1:**
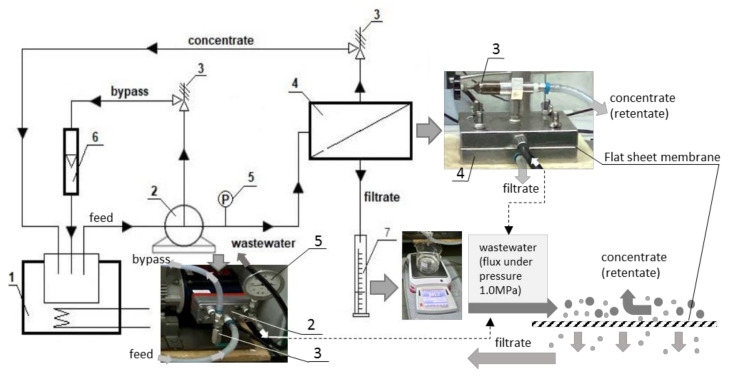
Schematic diagram of the nanofiltration system: 1—thermostat; 2—pump; 3—control valves; 4—pressure chamber with the membrane; 5—manometer; 6—rotameter; 7—measuring cylinder.

The nanofiltration process was carried out with the cross-flow method at a constant flow rate of liquid inside the bypass of 2 L·min^−1^ at 30 °C. The membrane filtration unit was built as model prototype by Danish Technological Institute (Taastrup, Denmark). The flow rate was regulated by rotameter (6). The flat sheet membranes were posted in a tight cylindrical pressure chamber (4). The tangential inlet of the feed caused a spiral flow of liquid in the pressure chamber (4). The pressure in the chamber (4) was regulated by two valves (3). The tests were performed at a pressure of 1.0 MPa. The thermostated feed solution was pumped from thermostat (1) through the pressure chamber. The permeate was collected in a separate vessel (7), which caused a gradual concentration of the feed stream (retentate) (Figure 1). The concentrate (retentate) was recycled to thermostat (1). The initial volume of the solution was 3 L. The solution was concentrated to a volume of 1.5 L (1:2). The measurements were taken at the beginning of the process (zero degree concentration), obtaining 0.5 L of filtrate from 3 L of the initial bath (concentration degree 1/6), and analogously—obtaining 1 L of filtrate (concentration degree 1/3) and 1.5 L of filtrate (concentration degree 1/2). The filtrate samples were taken directly with the hose draining the filtrate from the pressure chamber (4). At the same time, the retentate samples were taken from the thermostat (1).

Four flat sheet membranes with an area of approximately 314 cm^2^ each were selected for the tests, the parameters of which are summarized in Table 2. A new, previously unused membrane was used for each experiment. Before use, each membrane was subjected to a pressure of 1.5 MPa for 50 min at ambient temperature during the filtration of pure deionized water. After 50 min, the pressure was reduced to 1.0 MPa and the flow rate of the filtrate stream was measured for 10 min at 30 °C.

The experimental results are expressed in terms of the retention by the membrane of the compounds used in the present work. Retention is determined by employing the substance concentration in the permeate with reference to a representative value of the substance concentration in the retentate solution, as follows:

(1) $$R=\left(1-\frac{C_{P}}{C_{R}}\right)\cdot 100\%\;$$

where *C_P_* and *C_R_* are the concentrations of a specific compound in the permeate and retentate, respectively.

### 2.3. Analytical Procedures

All chemical compounds were monitored by determining their concentration using a Shimadzu Nexera-i LC-2040C 3D plus an apparatus equipped with a Kinetex C18 column (2.6 µm). The 0.1% formic acid–water solution (A) and the 0.1% formic acid–acetonitrile solution (B) were used as eluents. The columns were thermostated at 40 °C. The injection volume was 10 µL. Flow rates were from 0.4 to 0.5 mL·min^−1^.

A series of tests was performed in the natural water matrix from the Dobrzynka River (RW). The natural water samples were collected in September 2020 and stored at 6 °C. Table 3 presents the characteristic parameters of RW [31]. The total organic carbon (TOC), chemical oxygen demand (COD), and the selected ions were measured with using appropriate cuvette tests (HACH LANGE) and a photometer DR 3900 (HACH LANGE).

## 3. Results and Discussion

**Figure 2:**
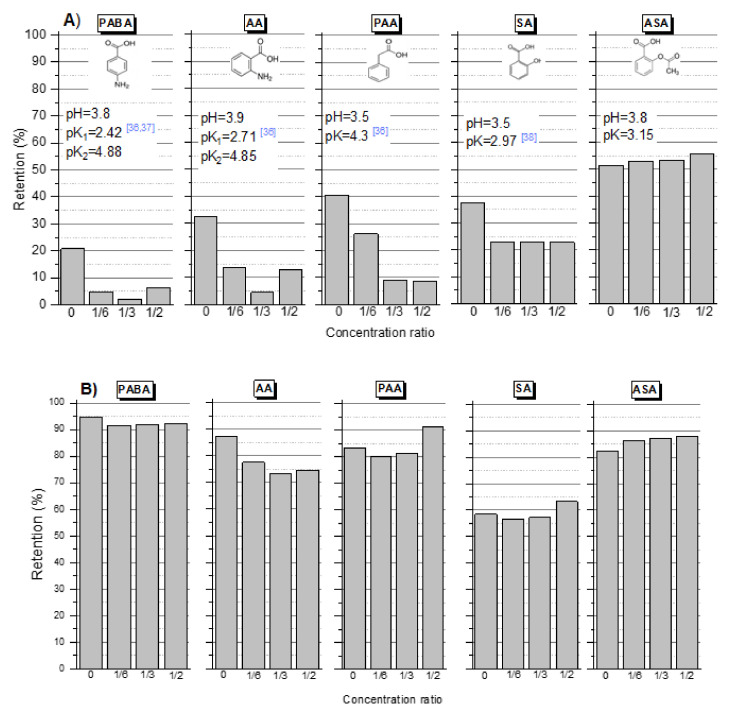
Retention value depending on the concentration ratio for selected compounds: (**A**) initial pH = 3.5–5; (**B**) pH = 8 (NaOH); membrane HL, pressure 1.0 MPa, temp. 30 °C [36,37,38].

**Figure 3:**
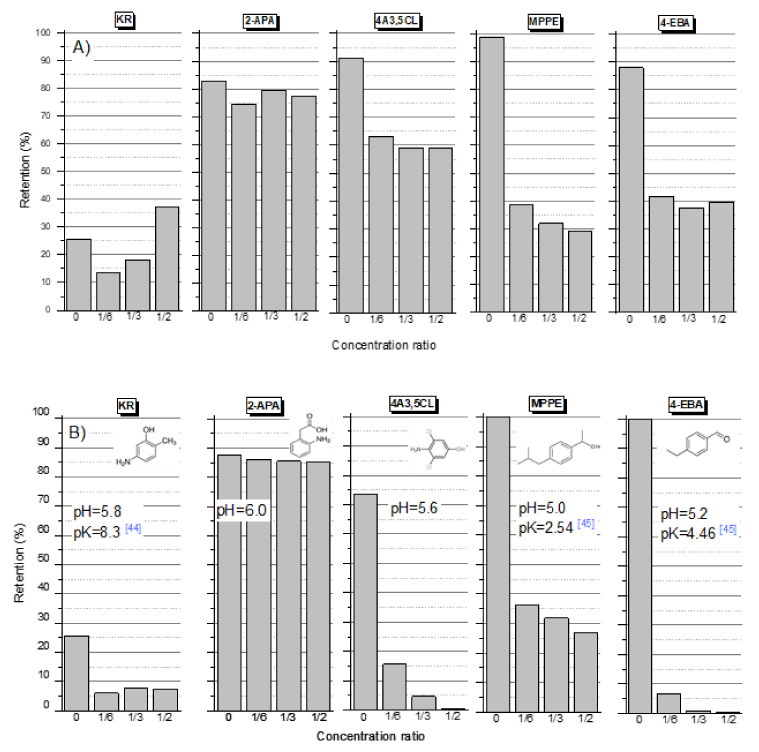
Retention value depending on the concentration ratio for selected compounds: (**A**) initial pH = 5–6; (**B**) pH = 8 (NaOH); membrane HL, pressure 10 bar, temp. 30 °C [44,45].

A series of tests were performed for various aromatic compounds with an amino group, including the oxidation products of the pharmaceuticals IBU and DCF. The pH values of the tested solutions ranged from three to six, depending on the type of the dissolved compound (pH and pKa values are presented in the Figure 2 and Figure 3). The tests were also performed at pH 8 (NaOH correction). Figure 2 and Figure 3 show the retention value depending on the concentration ratio for selected compounds. Figure 2 includes a group of compounds whose retention significantly increased at pH 8. These compounds contain a carboxyl group, (PABA, AA, PAA, SA and ASA). The additional carboxyl group in ASA increased retention. Presumably, the additional group causes a spatial barrier that facilitates the “hooking” of the ASA in the pores of a membrane. The presence of an amino group in the aromatic ring reduced the electronegativity of the carboxyl group attached to this ring (PABA, AA). Compounds containing, in addition to the carboxyl group, the amino group were very weakly retained on the membrane at low pH. Increasing the pH to 8 caused the dissociation of the carboxyl group and increased the electronegativity of the molecule (PABA, AA, Figure 2B). Similar results were obtained in the other works [32,33,34]. Xiong et al. (2015) reported that acetic acid rejection increased dramatically from 0% to 62% for DL and 0% to 60% for DK with the increase in pH from 3 to 7 [32]. Lactic acid rejection also increased significantly, from 49% to 84% for DL and 37% to 91% for DK, respectively. pH can affect both the surface charge of the membrane and the degree of ionization of the solutes, resulting in distinct solute transport effects. Wang et al. (2002), using two commercial nanofiltration membranes in permeation experiments with L-phenylalanine and L-aspartic acid aqueous solutions obtained rejections of about 0% and 90%, respectively, at pH values ranging between 4 and 9 [35].

Membranes with a polyamide polymer top layer have amide and carboxylic groups, which means that, depending on the pH, the membrane can be positively or negatively charged. Typically, at a pH of 3–4, the membranes have an isoelectric point and their zeta potential is close to zero. Above the pH at the isoelectric point, polyamide membranes are negatively charged, while below the pH they are positively charged. According to literature data, the HL membrane has an isoelectric point at pH = 3.3–3.7 [39,40]. The poor interaction of the molecules with the uncharged membrane results in low retention rates of the pollutants removed, especially in the case of compounds containing a positively charged amino group.

Figure 3 shows the dependence of retention on the concentration ratio for compounds for which the increase in pH did not affect the retention value (2-APA, MPPE) or had a negative effect (KR, 4A3,5CL, 4-EBA). KR and 4A3,5CL instead of a carboxyl group they have a hydroxyl group. The retention is probably influenced by the amino group. Lin et al. (2006) used a hybrid process of clay adsorption and ultrafiltration for removal of phenol and o-cresol (without amino group) from water. The rejection of o-cresol increased with increasing pH [41]. Sabaté et al., (2008) analyzed the ability of nanofiltration membranes to separate biogenic amines; feed pH strongly influenced solute rejection [42]. In acidic conditions up to pH 4, rejections of around 98, 96 and 90% were attained for histamine, putrescine and tyramine, respectively. Very low rejections of 5–10% were found at neutral pH and only moderate 20–40% rejections were generally observed in the alkaline zone.

The oxidation product of diclofenac 4A3.5CL, in addition to the hydroxyl and amine groups, has two substituted chlorine atoms. The Cl, O or Br in the organic molecule have the possibility of forming hydrogen bonds with the surface functional groups of the membrane (i.e., carboxyl, hydroxyl groups, and amine groups) due to their high polar nature [43].

There was an increase in the retention efficiency of the 2-APA compound (Figure 3A) relative to the PAA molecule (Figure 2A). The sodium salt of 2-APA molecule contained an additional amino group on the aromatic ring. The 2-APA was in the form of a sodium salt, the pH solution of which ranged between 5.5 and 6, while the pH of the PAA solution was approximately 3.5. This probably contributed to the greater influence of the electrostatic interaction between the membrane and the molecule. On the other hand, the presence of an amino group in the aromatic ring decreases the electronegativity of the carboxylic groups in the molecule. This may be the reason for the relatively little effect of the alkaline pH of the solution on the retention of 2-APA.

**Figure 4:**
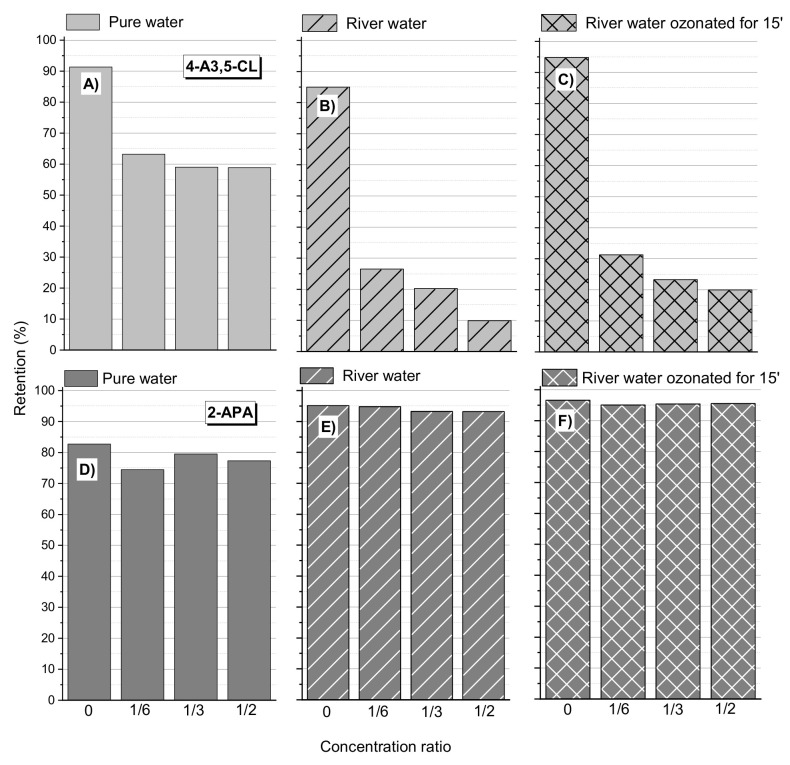
The influence of the water matrix on the retention of DCF oxidation products: (**A**) 4-Amino-3,5-dichlorofenol (4-A3,5-Cl) in pure water; (**B**) 4-A3,5-Cl in river water; (**C**) 4-A3,5-Cl in river water ozonated for 15 min (ozone dose in the gas phase: 31.5 mg O_3_ per L of water); (**D**) 2-Aminophenylacetic acid (2-APA) in pure water; (**E**) 2-APA in river water; (**F**) 2-APA in river water ozonated for 15 min (ozone dose in the gas phase: 31.5 mg O_3_ per L of water); membrane HL, pressure 1.0 MPa, 30 °C. See Table 1 for the composition of the river water matrix.

**Figure 5:**
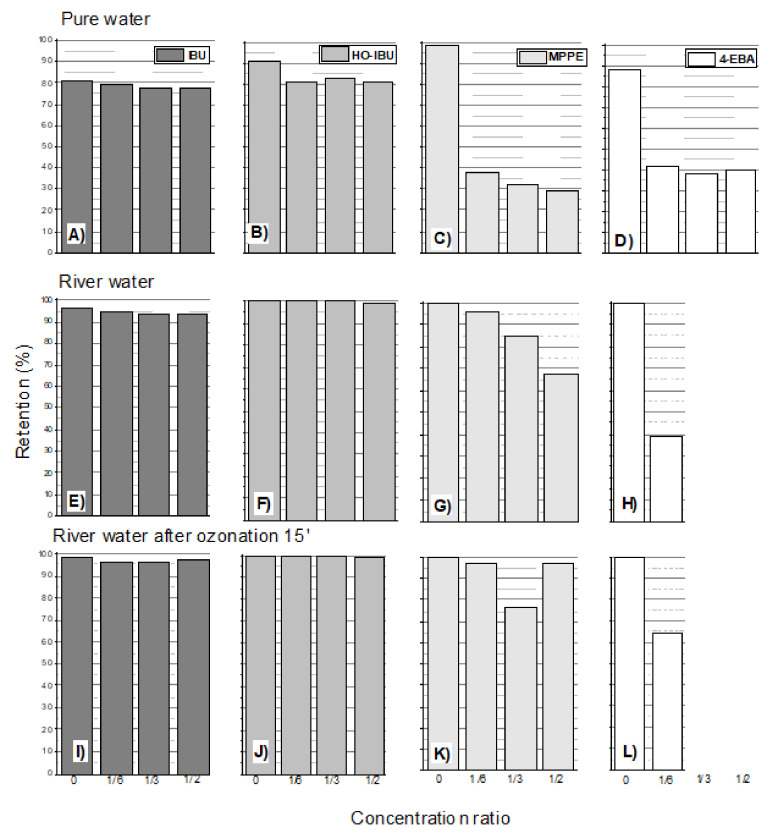
The influence of the water matrix on the retention of IBU oxidation products: (**A**) Ibuorfen (IBU) in pure water; (**B**) 41-hydroxyibuprofen (OH-IBU) in river water; (**C**) 1-(4-isobutyl-phenyl)ethanol (MPPE) in pure water; (**D**) 4-Ethylbenzaldehyde (4-EBA) in pure water; **E**) IBU in river water; (**F**) OH-IBU in river water; (**G**) MPPE in river water; (**H**) 4-EBA in river water; (**I**) IBU in river water ozonated for 15 min (ozone dose in the gas phase: 31.5 mg O_3_ per L of water); (**J**) OH-IBU in river water ozonated for 15 min (ozone dose in the gas phase: 31.5 mg O_3_ per L of water); (**K**) MPPE in river water ozonated for 15 min (ozone dose in the gas phase: 31.5 mg O_3_ per L of water); (**L**) 4-EBA in river water ozonated for 15 min (ozone dose in the gas phase: 31.5 mg O_3_ per L of water); membrane HL, pressure 1.0 MPa, temp. 30 °C. See Table 1 for the composition of the river water matrix.

The test results were compared with the experiments carried out for natural river water and river water ozonized for 15 min. The results for the DCF and IBU products are shown, respectively, in Figure 4 and Figure 5.

The river water contained natural organic matter (COD approx. 30 mg O_2_/L) and a relatively high pH 8. Theoretically, these factors usually increased the retention of the tested compounds [33,34,46]. Nghiem and Coleman (2008) noticed the significant enhancement in rejection of triclosan when the membranes were pre-fouled with the three model organic foulants, namely, bovine serum albumin (BSA), alginate and humic acid. Such a result was obtained for 2-APA (DCF product; Figure 4) and for the IBU products HO-IBU and MPPE (Figure 5). For 4A3,5-CL (DCF product), a significant deterioration in retention in the natural water matrix from the river was observed. Nevertheless, it seems that the presence of natural organic matter and other pollutants present in the river water did not significantly affect the 4-EBA retention. In all cases, it was observed that pre-ozonation of raw water from the river improved the retention of all tested compounds, including 4A3,5-CL. This can be explained by the fact that the ozonation process produces low-molecular-weight oxidation products (organic acids) that tend to adsorb and block the pores of the membrane [47,48].

**Figure 6:**
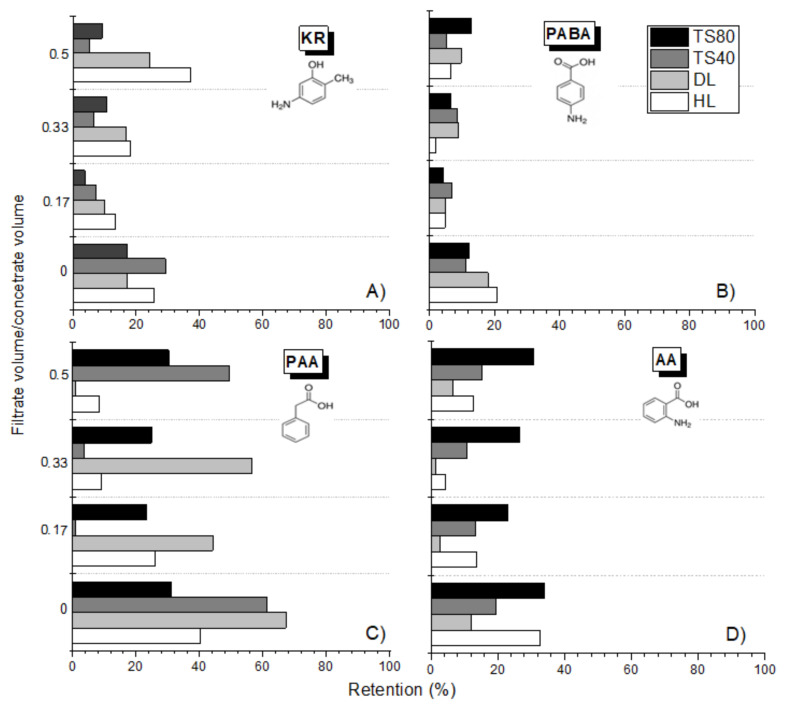
Dependence of retention on the concentration ratio (filtrate volume to concentrate volume ratio) for selected compounds for various types of commercial membranes: (**A**) 5-Amino-o-kresol (KR); (**B**) p-Aminobenzoic Acid (PABA); (**C**) Phenyloacetic Acid (PAA); (**D**) Anthranilic Acid (AA).

The experimental results were confirmed for the other commercial membranes, TS80, TS40, DL and HL (Figure 6). The zeta potential and the contact angle of the top layer of the membrane affect the separation efficiency. TS40 and TS80 membranes haD an isoelectric point (IEP) at pH = 2.5 [30], while the DL and HL membranes had a zero potential at pH = 3.3–3.7 [39,40,49]. At the pH where the membrane had a zeta potential close to zero, the electrostatic repulsion was lowest. The highest retention values were obtained for PAA (pH = 3.5) using the DL and TS40 membranes. According to the literature, these membranes are more electronegative than the HL membrane [30,39,40,49]. The lack of an amino group in the PAA molecule and the negative surface charge of the TS40 and DL membranes allowed for a relatively higher PAA retention compared to the HL and TS80 membranes. Although higher retentions would have been expected for the TS80 membrane due to the fact that it is an aromatic dense membrane [29]. Despite the similar molecular weight of PABA and AA, as well as the similar value of the pH solutions and their pKa, the retention of PABA was lower than that of AA. Probably, the substitution place of the amino group and carboxyl group in the aromatic ring had an effect.

In the case of KR, the highest retention values were obtained for the HL membrane, the least negatively charged HL [39,40,49]. This may indicate the influence of a positively charged amino group.

**Figure 7:**
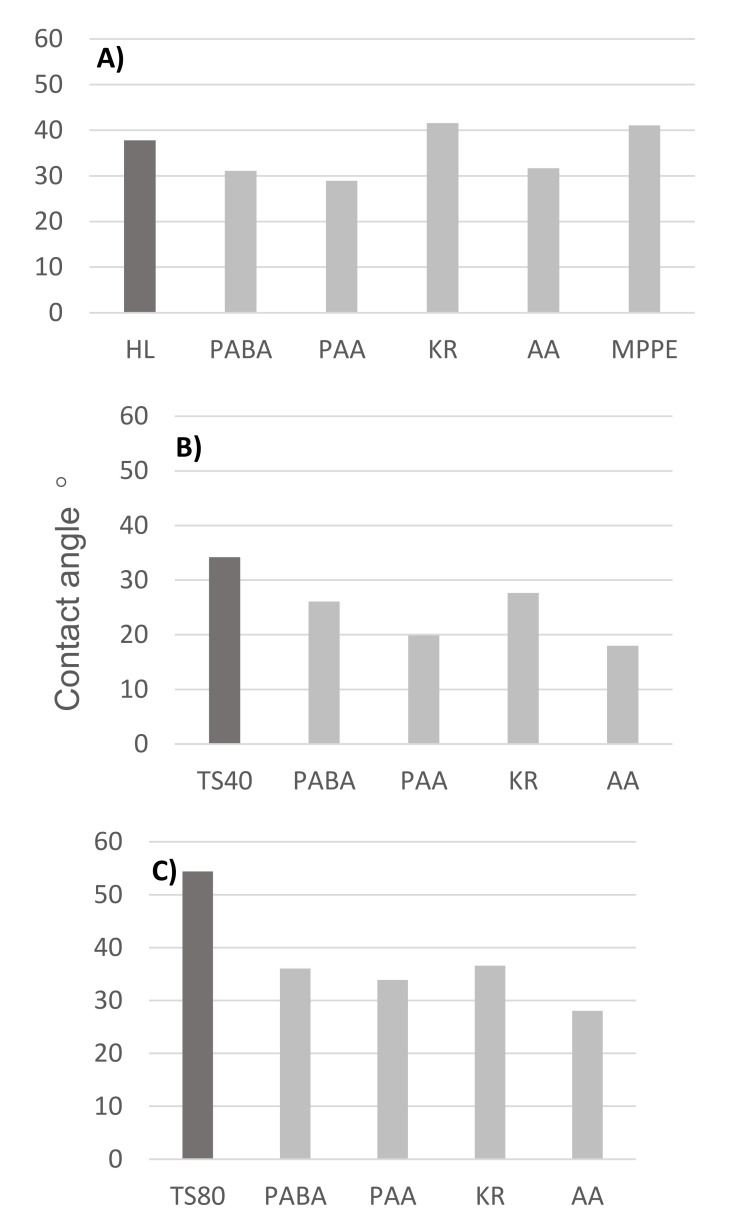
The values of the contact angle of the membranes (**A**) HL, (**B**) TS40 and (**C**) TS80 after the nanofiltration process for the selected compounds; dark gray bar—new, clean membrane; light gray bars—membranes after filtration process of PABA, PAA, KR and AA.

Figure 7 shows the values of the membrane contact angle after the nanofiltration process of the selected compounds PABA, PAA, KR, AA and MPPE. It should be emphasized that in addition to hydrophobicity, specific interactions may exist between amino acids and the membrane polymer [48].

Studies have shown that compounds such as KR and MPPE are hydrophobic and can increase the contact angle of hydrophilic membranes (Figure 7A, HL membrane). In the case of membranes with a higher contact angle (TS80 aromatic membrane), the adsorption of the selected compounds on the membrane resulted in a lower contact angle (Figure 7C). Although relatively smallest decrease in contact angle was noticed for the compounds MPPE and KR.

The retention of a given compound, in addition to the contact angle of the membrane surface, is influenced by other factors such as the charge of the membrane surface (expressed as zeta potential) and the pore size (expressed as MWCO value). Hence, different results were obtained for different membranes. In general, the lowest values of retention were obtained for PABA, which has an amino group in the para position to the carboxyl group. The higher values of retention were obtained with AA, which has an amino group in ortho position to the carboxyl group. It should be noted that, in the case of the TS80 membrane, no very marked differences were observed between the initial retention value and the in-process values, which was typical for an HL membrane. A clear decrease in retention during the filtration process usually indicates the dominant adsorption process in the separation mechanisms.

Aromatic amines and their degradation products may contribute to the formation of n-nitrosamines, the carcinogenicity of which has been confirmed. Further research is needed on the effectiveness of the removal of compounds with amino groups in membrane filtration processes. The low efficiency of the separation of these compounds in the wastewater treatment process may pose a serious threat to the environment and human health.

## 4. Conclusions

It was shown that the presence of the amino group and its position in relation to the carboxyl group in the aromatic ring affect the retention in the nanofiltration process. The experiments showed that the presence of an amino group in the molecule can significantly reduce the separation efficiency, even in the case of dense aromatic polymer membranes.

The oxidation products of some pharmaceuticals, such as 4-Amino-3,5-dichlorophenol—the oxidation product of diclofenac or 4-ethylbenzaldehyde—the product of IBU oxidation showed poor separation efficiency in the selected commercial membranes, regardless of the pH value and the presence of natural organic matter (NOM).

Further research is needed on the effectiveness of the removal of compounds with amino groups in membrane filtration processes. The low efficiency of the separation of these compounds in the wastewater treatment process may pose a serious threat to human health and the environment.

It has been shown that the pre-ozonation of natural river water can improve the retention of pollutants removed therefrom. It is supposed that this is due to the formation of low-molecular-weight oxidation products, which constitute an additional spatial barrier inside the pores of the membrane.

## Figures and Tables

**Table 1:** Structural formulas of selected compounds.

Name	Salicylic Acid (SA)	Acetylsalicylic Acid (ASA)	p-Aminobenzoic Acid (PABA)
Chemical structure	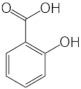	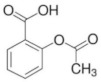	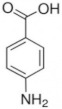
CAS number	69-72-7	50-78-2	150-13-0
Molar mass (g·mol^−1^)	138.12	180.16	137.14
**Name**	**Phenyloacetic Acid** **(PAA)**	**5-Amino-o-kresol** **(KR)**	**Anthranilic Acid** **(AA)**
Chemical structure	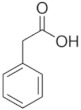	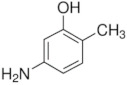	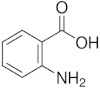
CAS number	103-82-2	2835-95-2	118-92-3
Molar mass (g·mol^−1^)	136.15	123.15	137.14
**Name**	**4-Amino-3,5-dichlorofenol (4-A3,5-Cl)** ***Diclofenac oxidation product***	**2-Aminophenylacetic acid (2-APA)** ***Diclofenac oxidation product sodium salt***	**Ibuprofen (IBU)** **Sodium salt**
Chemical structure	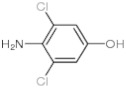	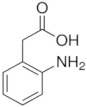	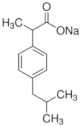
CAS number	26271-75-0	3342-78-7	31121-93-4
Molar mass (g·mol^−1^)	178.0	174.16	228.26
**Name**	**1-hydroxyibuprofen** (OH-IBU) ***Ibuprofen oxidation product***	**4-Ethylbenzaldehyde** **(4-EBA) *Ibuprofen oxidation product***	**1-(4-isobutyl-phenyl)ethanol (MPPE) *Ibuprofen oxidation product***
Chemical structure	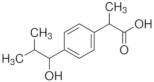	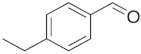	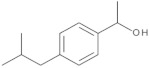
CAS number	53949-53-4	4748-78-1	40150-92-3
Molar mass (g·mol^−1^)	222.28	134.18	178.27

**Table 2:** Parameters of membranes used in the study.

Characteristic	Type of Membrane			
	HL	DL	TS40	TS80
polymer	piperazine polyamide [27,28]	modified piperazine polyamide [29]	piperazine polyamide [30]	aromatic polyamide [30]
pH range	3–9	2–10	2–11	2–11
MCWO (Da)	150–300	~150–300	~200	~150
retention MgSO_4_/NaCl	98.0%/n.d.	98%/n.d.	90.0%/40–60%	99.0%/80–90%
hydraulic permeability filtrate flux (Lm^−2^·h^−1^)/Pressure (MPa)	66/0.69	48/1.52	32/0.76	32/0.76
manufacturer	GE Osmonics	GE Osmonics	TriSep^TM^	TriSep^TM^

**Table 3:** Characteristic parameters of natural river water (RW) [31].

Parameter	Value	Standard Deviation
pH	8.06	0.007
conductivity, µS·cm^−1^	570	0.71
COD, mg·L^−1^	30.7	0.07
TOC, mg·L^−1^	10.2	0.14
K^+^, mg·L^−1^	4.66	0.25
PO_4_^3−^, mg·L^−1^	0.148	0.01
SO_4_^2−^, mg·L^−1^	43	1.56
NO_3_^−^, mg·L^−1^	1.73	0.08
Cl^−^, mg·L^−1^	31.3	1.77
CO_2_, mg·L^−1^	162	2.12

## Data Availability

The data presented in this study are available on request from the corresponding author.
